# Inflammatory biomarkers and oral microbiome alterations in depression and anxiety disorders: a systematic review

**DOI:** 10.1080/15622975.2026.2688864

**Published:** 2026-07-09

**Authors:** Alec Juhl, Sun Ha Park, Mitra Simanian, Yan Wang

**Affiliations:** a UCLA School of Dentistry, Los Angeles, California, USA; b Faculty, UCLA School of Dentistry, Los Angeles, California, USA

**Keywords:** Oral microbiome, depression, anxiety disorders, inflammatory biomarkers, psychoneuroimmunology

## Abstract

**Background/Objectives:**

Depression and anxiety are increasingly linked to systemic inflammation and microbiome alterations, yet the role of the oral microbiome remains poorly characterised. This systematic review synthesises recent human evidence examining associations between depression or anxiety and (1) peripheral or salivary inflammatory biomarkers and (2) oral microbiome alterations.

**Materials and Methods:**

Following PRISMA 2020 guidance, PubMed, Web of Science, and PsycINFO were searched for studies published between 2016 and 2026. Eligible studies assessed depression, depressive symptoms, anxiety, generalised anxiety disorder (GAD), or PTSD-related symptoms alongside inflammatory biomarkers in blood or saliva and/or oral microbiome profiles. Reference lists of key eligible studies were also screened. Risk of bias was assessed using the Newcastle-Ottawa Scale (NOS) or an adapted NOS framework.

**Results:**

Fifty-three primary studies met eligibility criteria, including 42 studies evaluating inflammatory or salivary biomarkers and 11 studies examining oral microbiome profiles. Depression was associated with alterations in pro-inflammatory markers, particularly CRP, IL-6-related signalling, TNF-α, and other cytokine or chemokine markers. Anxiety-related findings were more heterogeneous. Oral microbiome studies reported altered community composition and taxa associated with depression, anxiety, and trauma-related symptoms, but findings varied by population, sampling site, and adjustment for oral-health and behavioural confounders.

**Conclusions:**

Current evidence suggests depression and anxiety-related conditions are associated with low-grade inflammatory activity and alterations in the oral microbiome. These findings support an oral-immune-brain framework for future research, but the current evidence remains largely observational.

## Introduction

Depression and anxiety are prevalent psychiatric disorders characterised by mood dysregulation, psychological distress, and significant impairment. Beyond their neurochemical underpinnings, these disorders have been increasingly associated with alterations in immuneinflammatory activity. Clinical and epidemiological studies over the past two decades have converged on the finding that a subset of individuals with major depressive disorder (MDD) exhibit elevated levels of pro-inflammatory biomarkers in peripheral blood (Osimo et al. 2020; Kappelmann et al. 2021). Concentrations of C-reactive protein (CRP) and interleukin-6 (IL-6) in particular are often higher in patients with depression compared to non-depressed controls (Osimo et al. 2020; Ye et al. 2021). Meta-analyses indicate that other cytokines – including IL-1β, TNF-α, IL-12, and IL-18 – are also upregulated on average in depression, alongside acute-phase reactants like CRP (Dowlati et al. 2010; Osimo et al. 2020). Collectively, this body of evidence has given rise to the “cytokine hypothesis” of depression, which proposes that excess peripheral inflammation can induce neurobiological changes contributing to depressive symptoms (e.g. *via* cytokine effects on neurotransmitters and the HPA axis).

Anxiety disorders have received comparatively less focus in inflammation research, but interest is growing in their immunological aspects. Depression and anxiety frequently co-occur, and some immune findings appear overlapping. For example, generalised anxiety disorder (GAD) has been associated with elevated CRP and certain cytokines in population studies (Costello et al. 2019; Ye et al. 2021). However, results have been mixed, with some studies showing minimal or no inflammatory differences in pure anxiety states after accounting for depression comorbidity (Shen et al. 2022). PTSD-related symptoms are also relevant to this review because trauma exposure and post-traumatic symptoms share affective and inflammatory features with anxiety-related conditions; however, PTSD is classified in DSM-5/DSM-5-TR as a trauma- and stressor-related disorder rather than an anxiety disorder, and PTSD findings are interpreted separately when relevant (American Psychiatric Association 2022).

In parallel with immune research, the past decade has witnessed a surge of interest in the role of the microbiome in psychiatry. While most studies have centred on the gut microbiome, recent work has begun to explore other microbial niches. The oral cavity harbours one of the most diverse microbiomes in the body and is a gateway to both the gastrointestinal and respiratory tracts. Oral bacteria can contribute to systemic inflammation – for instance, through periodontal disease, which has been linked to elevated circulating inflammatory markers and even atherosclerosis. It stands to reason that disturbances in the oral microbiome (“oral dysbiosis”) might have implications for systemic health and possibly neuropsychiatric outcomes. Indeed, oral health has been observed to correlate with mental health; patients with severe mental illness often have poorer dental health, and chronic stress can alter saliva composition and immunity in the mouth. Despite this, the “oral microbiome–brain axis” remains relatively under-studied. Only recently have researchers applied varying sequencing techniques to investigate whether the oral microbial community differs in individuals with depression or anxiety.

The rationale for examining the oral microbiome in this context is multifold. First, certain oral bacteria could translocate or release inflammatory components (e.g. endotoxin) that stimulate systemic immune responses, thereby affecting the brain. Second, dysbiosis in the oral cavity might reflect chronic activation of inflammatory pathways that are also relevant in depression/anxiety pathophysiology. Third, identifying consistent microbial biomarkers in saliva could pave the way for non-invasive screening tools for mental health risk, given saliva is easily obtained. Early studies have reported, for example, that depressed individuals show shifts in oral bacterial composition, with preliminary findings of differences in genera like *Neisseria* and *Prevotella* (Wingfield et al. 2021). Since *Neisseria* species are generally regarded as normal commensals linked to oral health (some even help regulate nitric oxide), an observed decrease in these bacteria in depression could indicate a less healthy oral ecosystem (Agranyoni et al. 2025). On the other hand, some studies paradoxically found *Neisseria* higher in depression (Wingfield et al. 2021), highlighting that findings need to be contextualised and replicated. For anxiety, a large community study recently suggested specific oral microbial signatures associated with PTSD and anxiety symptoms (e.g. lower abundance of *Neisseria elongata* and higher *Oribacterium* in those with anxiety disorders) (Malan-Müller et al. 2024).

In summary, converging evidence suggests that two peripheral systems – the immune system and the oral microbiome – may be altered in people with depression and anxiety. However, these literatures remain in early stages, especially for the oral microbiome, and a synthesis of recent human findings is needed. To our knowledge, no prior systematic review has jointly examined inflammatory markers and the oral microbiome in relation to depression and anxiety using an updated search that includes PsycINFO. Therefore, we undertook a systematic review of human studies published from 2016 onward that investigated: (1) peripheral or salivary inflammatory biomarkers in relation to depression, depressive symptoms, anxiety, GAD, or PTSD-related symptoms; and (2) oral microbiome differences in relation to these psychiatric outcomes. Our goals were to summarise the magnitude and consistency of associations, highlight key biomarkers and microbial taxa identified, and discuss potential mechanisms and limitations linking these peripheral factors with mood and anxiety-related disorders.

## Methods

### Search strategy and selection criteria

This systematic review was conducted in accordance with PRISMA 2020 guidance (Page et al. 2021). We searched PubMed, Web of Science, and PsycINFO for English-language human studies published between January 1, 2016 and January 1, 2026. Because this review spans two related bodies of literature, searches were performed using concept-specific combinations of psychiatric terms, inflammatory or salivary biomarker terms, and oral microbiome terms. Psychiatric search terms included “depression,” “major depressive disorder,” “depressive symptoms,” “anxiety,” and “generalized anxiety disorder.” Biomarker search terms included “inflammatory biomarkers,” “salivary biomarkers,” “salivary cytokines,” “cytokines,” “interleukin,” “C-reactive protein,” “CRP,” and “TNF alpha.” Oral microbiome terms included “oral microbiome,” “oral microbiota,” “salivary microbiome,” and “oral bacteria.” Search strategies were adapted to each database interface using database-specific keyword queries and search fields. Exact search terms entered, search fields, filters, dates, record counts, and search links are provided in Supplementary Table S1. In addition to database searching, citation/reference screening of key eligible studies and relevant reviews was performed to identify additional eligible primary studies.

Records retrieved from all database searches were exported, combined, and de-duplicated before screening. Titles and abstracts were screened for relevance, followed by full-text review of potentially eligible studies. Studies were included if they were original human research studies that assessed depression, depressive symptoms, anxiety, GAD, or PTSD-related symptoms using a clinical diagnosis or validated symptom measure and examined inflammatory biomarkers in blood or saliva and/or oral microbiome composition. Review articles, meta-analyses, editorials, commentaries, case reports, animal studies, *in vitro* studies, non-English studies, and studies without relevant psychiatric or biological outcomes were excluded from the included-study dataset.

### Data extraction and quality assessment

Using a standardised extraction form, we extracted key data from each included study, including author(s), year, study design, sample characteristics, psychiatric assessment method, biological sample type, inflammatory biomarkers or microbiome methods, main findings, and reported covariate adjustment. For inflammatory biomarker studies, extracted markers included CRP, IL-6, TNF-α, interleukins, chemokines, inflammatory ratios, and other immune-related markers. For oral microbiome studies, we extracted sample type, sequencing or profiling method where reported, diversity findings, differentially abundant taxa, and reported functional pathway analyses. Information on key confounders, including age, sex, BMI, smoking status, psychotropic medication use, periodontal disease, oral hygiene behaviours, and antibiotic exposure, was extracted when available.

Risk of bias was assessed using the Newcastle–Ottawa Scale (NOS) for cohort and case-control studies and an adapted NOS framework for cross-sectional studies. Studies were evaluated across selection, comparability, and outcome/exposure domains. Particular attention was given to confounders relevant to inflammatory and oral microbiome outcomes, including age, sex, BMI, smoking status, psychotropic medication use, periodontal disease, oral hygiene behaviours, and antibiotic exposure. Studies scoring 7–9 were classified as low risk of bias, 4–6 as moderate risk of bias, and 0–3 as high risk of bias. The adapted scoring rubric used for cross-sectional studies is provided in Supplementary Table S2.

### Data synthesis

We first summarise results for inflammatory biomarkers and depression/anxiety, then results for the oral microbiome and depression/anxiety. Within each section, we distinguish findings for depression and for anxiety, but given the frequent comorbidity and overlap. PTSD-related findings are included because trauma-related symptoms overlap clinically and biologically with anxiety and depression, but PTSD is not treated as an anxiety disorder. Given the substantial heterogeneity in psychiatric definitions, biomarker assays, microbiome sampling methods, covariate adjustment, and outcome reporting, meta-analysis was not performed. Instead, findings were narratively synthesised by biological domain and psychiatric phenotype. Summary tables consolidate key biomarkers and oral microbiome findings associated with depression and/or anxiety-related symptoms.

## Results

### Study selection

**Figure 1.**
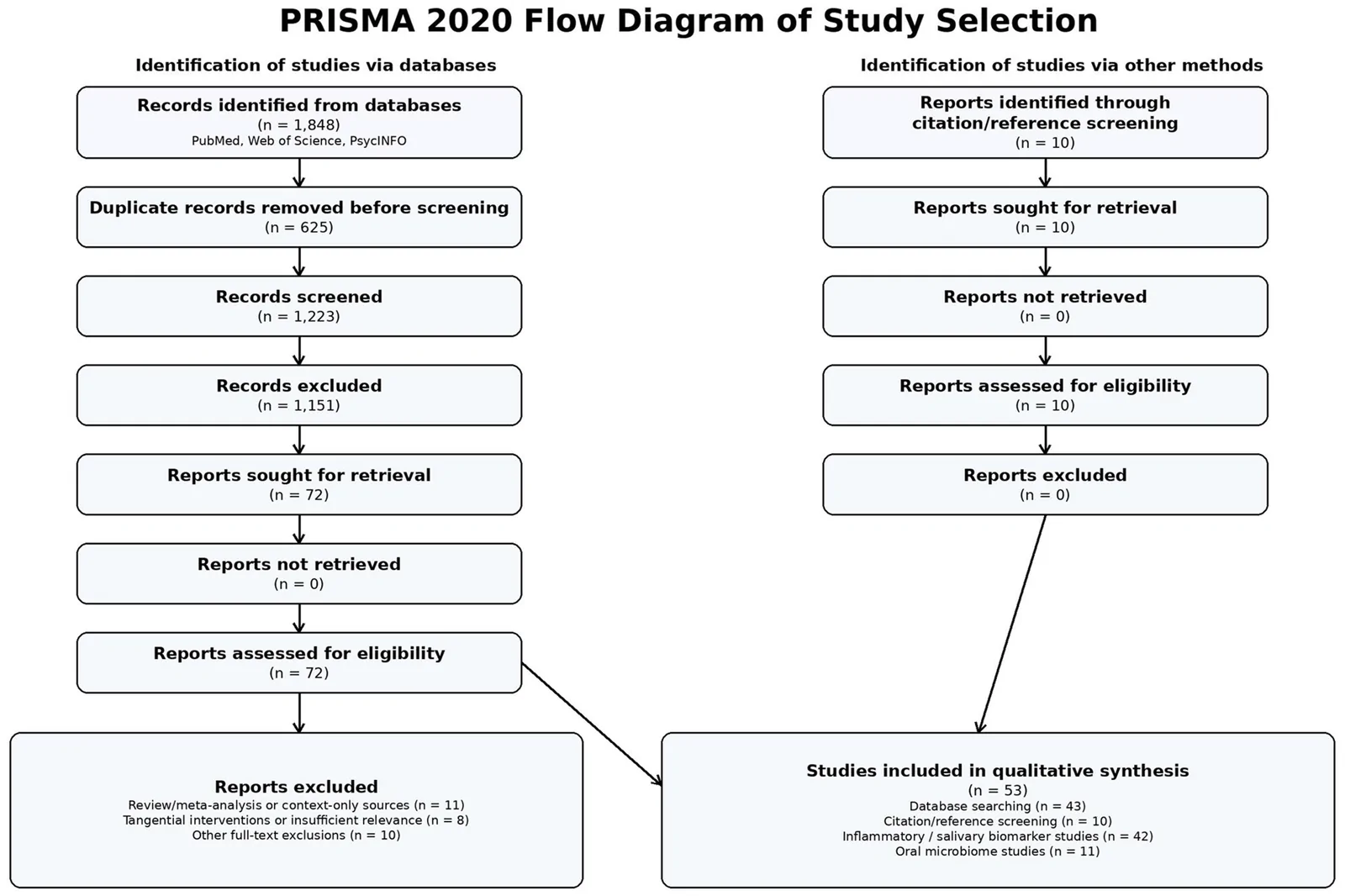
PRISMA 2020 flow diagram showing database searching, citation/reference screening, full-text eligibility, and included-study counts. The qualitative synthesis included 53 primary studies: 43 identified through database searching and 10 identified through citation/reference screening.

The updated database searches yielded 1,848 records before de-duplication. After removal of 625 duplicates, 1,223 unique database records were screened by title and abstract, and 1,151 were excluded at this stage. Seventy-two reports from database searching underwent full-text assessment, of which 29 were excluded after full-text review. Citation/reference screening identified 10 additional reports for full-text assessment, all of which met the eligibility criteria. In total, 53 primary studies were included in the qualitative synthesis: 43 identified through database searching and 10 through citation/reference screening. The study selection process is illustrated in Figure 1.

Of the 53 included studies, 42 investigated inflammatory or salivary biomarkers in relation to depression, anxiety, GAD, or PTSD-related symptoms, and 11 investigated the oral microbiome. The studies spanned general community samples, clinically diagnosed MDD and GAD samples, adolescents, pregnancy cohorts, trauma-exposed populations, and population-based cohorts. Most were cross-sectional or case-control studies, although several cohort or prospective studies provided longitudinal evidence.

Key peripheral inflammatory biomarkers associated with depression and anxiety are summarised in Table 1. Key oral microbiome taxa and diversity patterns associated with depression and anxiety are summarised in Table 2.

### Characteristics of included studies

The 42 inflammation- or salivary biomarker-focused studies encompassed a wide range of study designs and sample sizes, including several large cohort studies and smaller case-control studies. Most inflammation studies used blood samples (serum or plasma) to measure cytokines, chemokines, acute-phase proteins, and related immune markers, while a smaller subset measured salivary inflammatory markers. Depression was assessed *via* clinical diagnosis in some studies and *via* validated rating scales in others. Anxiety assessments varied and included GAD diagnosis, GAD symptom scales, and broader anxiety measures. Salivary inflammatory biomarker studies included salivary cytokine and salivary CRP analyses in MDD and trauma-exposed or PTSD-related samples (Yui et al. 2022; Robles et al. 2024).

The 11 oral microbiome studies primarily used saliva or oral samples and sequencing-based methods to characterise bacterial communities. Analytic approaches included alpha and beta diversity, differential abundance testing, and, in some studies, machine learning or predicted functional pathway analysis. Most controlled for basic covariates such as age and sex, but fewer studies systematically addressed smoking, medication use, periodontal disease, antibiotic exposure, oral hygiene, or xerostomia, which are important potential confounders for oral microbiome interpretation.

Intervention studies were not included unless baseline or clearly extractable observational associations were reported. Review articles and meta-analyses were used only for background or contextual discussion and were not counted as included studies.

### Associations between depression and anxiety and inflammatory biomarkers

Across the reviewed studies, depression was more consistently associated with inflammatory biomarker alterations than anxiety alone, although effect sizes and clinical relevance varied across studies. We first summarise findings for depression, then for anxiety-related outcomes, noting frequent comorbidity and overlap.

### Depression and inflammatory markers

Nearly all included studies on depression reported at least one inflammatory marker associated with depressive diagnosis, severity, or symptom domain, although the magnitude and clinical meaning of these differences varied. The two most frequently discussed biomarkers were CRP and IL-6. For example, Nishuty et al. (2019) found drug-naïve MDD patients had statistically higher serum IL-6 than healthy controls, but the absolute difference was modest and should be interpreted cautiously rather than assumed to indicate clinical significance. CRP was not significantly different on average in that study, though both IL-6 and CRP correlated positively with depression severity. Larger studies and cohort analyses have linked CRP, IL-6-related signalling, and inflammatory burden to depression or depressive symptoms, but the evidence supports heterogeneity rather than a uniform inflammatory profile across all depressed individuals (Kappelmann et al. 2021; Ye et al. 2021; Zainal and Newman 2021; Shi et al. 2022). Additional depression biomarker studies reported associations involving CRP, GlycA, FAM19A5, IL-6, and related inflammatory markers across cohort, clinical, and symptom-based samples (Schmidt et al. 2016; Jeenger et al. 2017; Köhler-Forsberg et al. 2017; Purnichi et al. 2019; Brunoni et al. 2020; Han et al. 2020; Manfro et al. 2022; Foley et al. 2024; Martino et al. 2025).

Importantly, longitudinal studies support that inflammation may precede or predict depression in some cases, although these associations remain observational. For example, population-based prospective work has shown that inflammatory markers can predict later depressive symptoms or diagnostic changes over time (Zainal and Newman 2021; Shi et al. 2022). These findings align with the concept of a “primed” inflammatory response contributing to depression in vulnerable individuals, but they do not prove a single causal pathway.

Beyond IL-6 and CRP, other cytokines and immune-related markers have been linked to depression in recent primary studies. Reported markers include TNF-α, IL-1β, IL-2, IL-7, IL-10, IL-12, IL-18, IL-33, chemokines, complement-related markers, and suppressors of cytokine signalling. Several studies in drug-naïve or unmedicated MDD samples suggest immune alterations, but the direction, magnitude, and clinical meaning of these findings vary by population, symptom profile, medication status, and analytic method (Tang et al. 2021; Gao et al. 2022; Li et al. 2022; Xu et al. 2023; Guo et al. 2024). Other included primary studies examined IL-7/IL-10, IL-1β/TNF-α, IL-18, suppressors of cytokine signalling, peripheral blood-cell inflammatory markers, cytokine-related genes, and specific cytokine/chemokine profiles in depression (Fan et al. 2017; Anjum et al. 2020; Buspavanich et al. 2021; Das et al. 2021; Kobayashi et al. 2022; Min et al. 2023; Nayem et al. 2023; Nahar et al. 2023a; Nahar et al. 2023b; Nahar et al. 2023c; Liang et al. 2024; Liu et al. 2024; Oh et al. 2025).

An emerging theme is that depression’s inflammatory signature may be symptom-specific. Ye and colleagues performed a granular analysis linking CRP/IL-6 to particular symptom domains in ~145k UK Biobank participants (Ye et al. 2021). It was found that higher CRP was associated especially with somatic symptoms of depression – such as sleep disturbance, fatigue, and appetite changes – more so than with pure psychological symptoms (Kappelmann et al. 2021). IL-6 was similarly associated with these energy-related symptoms (and with low mood to some extent) (Kappelmann et al. 2021). Anxiety symptoms showed weaker and less consistent links to IL-6/CRP in that analysis. This aligns with other reports that patients with so-called “atypical” depression (characterised by hypersomnia and hyperphagia) or high fatigue tend to have elevated inflammatory markers (Kappelmann et al. 2021). It raises the possibility that inflammation contributes preferentially to certain depression phenotypes, which has implications for targeted treatments (e.g. anti-inflammatory therapy might particularly help those with high inflammation and somatic symptoms).

### Anxiety and inflammatory markers

Findings for anxiety disorders and inflammation are more variable. Several studies found that when depression is controlled for, the association between inflammation and anxiety attenuates or disappears (Kappelmann et al. 2021; Ye et al. 2021). That said, certain anxiety-related populations do show inflammatory differences. Studies of GAD have reported altered CRP, IL-6, IL-4, IL-10, IL-12, IL-17A, IL-23A, TNF-α, MCP-4, S100B, and related cytokine markers, but directionality is inconsistent and may depend on comorbidity, medication status, sample age, and study design (Shen et al. 2022; Mamun-Or-Rashid et al. 2024; Sarmin et al. 2024; Alfi et al. 2025). Population-based and prospective studies also linked anxiety disorders or GAD with CRP, IL-6, adiponectin, or inflammatory symptom patterns, although directions were inconsistent (Naudé et al. 2018; Lee 2020; Wagner et al. 2020).

Interestingly, Shen et al. (2022) challenged the notion of pro-inflammatory profiles in GAD. They measured a panel of cytokines and S100B in GAD patients and controls and found lower IL-1β and IL-2, higher IL-4, and no significant difference in IL-10. These findings suggest that anxiety disorders are heterogeneous and may not share the same inflammatory pattern as depression.

Another perspective is offered by Mendelian randomisation analyses. Large population-based work suggests that CRP and IL-6-related signalling may relate differently to depression and anxiety, and genetically informed analyses do not support a simple linear causal interpretation for all inflammatory markers. Thus, while anxiety symptoms can correlate with inflammation because of overlap with depression or shared stress exposure, current evidence does not establish inflammation as a primary driver of anxiety in the absence of depression (Kappelmann et al. 2021; Ye et al. 2021).

In summary, depression is associated with inflammatory alterations in a subset of patients, whereas anxiety disorders show a less consistent pattern, with evidence of inflammation mainly in contexts of comorbidity, chronic stress, or trauma-related symptoms. This overlap makes it challenging to ascribe effects to “anxiety only.” The clearest signal for anxiety-related inflammation may occur in stress- or trauma-related populations, although PTSD should be discussed separately because it is classified as a trauma- and stressor-related disorder rather than an anxiety disorder.

Anti-inflammatory treatments are being explored as adjunctive approaches for depression, but treatment trials were not the focus of the present review. The biomarker evidence synthesised here provides rationale for future stratified studies, particularly among patients with elevated inflammatory markers at baseline. For anxiety, inflammation-targeted approaches remain less developed and may be most relevant when anxiety co-occurs with depression, trauma exposure, or inflammatory medical burden.

### Associations between depression and anxiety and the oral microbiome

Research on the oral microbiome in mental health is still emerging, but the included studies suggest that depression and anxiety-related symptoms are associated with shifts in the oral microbial ecosystem. The reported changes involve both broad community characteristics, such as diversity and composition, and specific bacterial taxa, although findings are not fully consistent across populations. Additional oral microbiome studies included adolescent, population-based, NHANES-based, male cohort, oral-pathogen, and host-epigenetic analyses (Simpson et al. 2020; Girella et al. 2025; Kerff et al. 2025; Liu et al. 2025; Qiu et al. 2025; Zheng et al. 2025).

### Depression and the oral microbiome

Multiple independent studies suggest that individuals with depression have a distinct salivary microbiome compared to non-depressed controls. For example, Wingfield et al. (2021) conducted one of the first case–control studies on this topic, examining young adults (mean age ~21) with MDD versus healthy peers. They observed significant differences in both alpha and beta diversity: the overall microbial communities clustered differently between depressed and healthy individuals (Wingfield et al. 2021). Notably, they identified 21 bacterial taxa that were differentially abundant, with the majority (19 taxa) showing *reduced* abundance in depressed participants (Wingfield et al. 2021). However, a subset of taxa were enriched in depression, including increased *Neisseria* (spp.) and *Prevotella* nigrescens (Wingfield et al. 2021). *Neisseria* are typical oral commensals, whereas *Prevotella nigrescens* is associated with periodontal disease. The authors interpreted the overall pattern as a subtle dysbiosis: depression was accompanied by loss of many normal microbes (hence lower abundance of most taxa) and a relative bloom of a few organisms (some potentially pathogenic) (Wingfield et al. 2021). This was a preliminary study (*n* ≈ 40 per group), but it pioneered the idea of an “oral microbiome signature” of depression.

Subsequent studies have both corroborated and nuanced these findings. In a pregnant population, Agranyoni et al. (2025) found that women with clinically significant depressive symptoms had lower abundance of several taxa, including Neisseria, compared with women without depressive symptoms. This contrasted with the direction reported in some young adult samples, suggesting that context such as age, pregnancy, oral health, and hormonal or immune status may influence which microbes differ in association with depressive symptoms.

An adolescent depression study by Zeng et al. (2025) adds further evidence. In this case–control study of 53 depressed teens (treatment-naïve) and 53 controls, depressed adolescents had significantly different oral microbiota composition, including differences in Streptococcus, Neisseria, Haemophilus, Fusobacterium, and an unclassified family of Bacteroidetes (Zeng et al. 2025). Using random forest classification, they found the top 10 genera that distinguished depressed youth from controls, achieving an AUC of 0.78 (Zeng et al. 2025). Among these top genera were *Streptococcus, Prevotella, Alloprevotella, Porphyromonas,* and *Fusobacterium* (many being known gingival bacteria), as well as *Neisseria* and *Haemophilus*. This suggests depression in adolescence may coincide with a pattern hinting at poorer periodontal health or altered immunity. They also found correlations between some of these oral bacteria and cognitive function scores (memory, etc.), implying that oral microbiota differences might not only reflect mental health status but could conceivably relate to cognitive or neurological outcomes (Zeng et al. 2025). The authors proposed the oral microbiota as a potential biomarker for adolescent depression (Zeng et al. 2025), though they cautioned that causality is unproven.

Synthesising across these studies, a few common themes for depression emerge:

- **Altered diversity:** Depression is associated with a change in diversity (sometimes lower, sometimes higher, likely depending on which taxa increase or decrease). It indicates a departure from the normative oral ecosystem.
- **Increases in certain bacteria with pro-inflammatory or dysbiotic potential:** e.g., *Prevotella*, *Fusobacterium*, and in some cases *Streptococcus* and *Porphyromonas*. These genera are often implicated in gum disease or systemic inflammation. Their higher abundance could signal that depression might lead to behavioural changes (like neglect of oral hygiene) or physiological changes (like altered saliva flow or composition) that favour these bacteria.
- **Decreases in potentially beneficial commensals:** e.g., *Neisseria* (in at least some populations) and possibly *Haemophilus* or *Capnocytophaga* (one study noted lower *Capnocytophaga* in those with depression and poor dental health) (Malan-Müller et al. 2024). If depression reduces saliva production via antidepressant medications or causes mouth breathing, that could also shift which bacteria dominate (since saliva and oxygen availability shape the oral microbiome).
- **Functional changes:** Malan-Müller et al. (2024) reported predicted functional pathways related to tryptophan metabolism in association with mental health symptoms. However, this should be interpreted as a hypothesis-generating correlation rather than evidence that oral bacteria directly reduce systemic tryptophan availability or plasma serotonin. Current human evidence does not establish a causal mechanism linking oral tryptophan metabolism to serotonin changes in depression or anxiety.

### Anxiety/PTSD and the oral microbiome

Though fewer studies have looked specifically at anxiety, some included research evaluated anxiety and trauma-related symptoms in conjunction with oral microbiota. The largest such study (Malan-Müller et al. 2024) profiled 470 individuals in a community sample with varying levels of depression, anxiety, and PTSD symptoms (Malan-Müller et al. 2024). They found that periodontal and mental health factors both influenced the oral microbiome (Malan-Müller et al. 2024). Focusing on anxiety: participants with a self-reported anxiety disorder diagnosis had a distinct microbiome signature – notably lower Neisseria elongata and higher Oribacterium asaccharolyticum relative to those without anxiety (Malan-Müller et al. 2024). Additionally, PTSD symptoms were correlated with specific changes (lower Haemophilus sputorum, higher Prevotella histicola), (Malan-Müller et al. 2024) and interestingly that Prevotella histicola was also associated with higher depression scores in the same sample (Malan-Müller et al. 2024). This suggests some overlap between depression and PTSD in terms of which bacteria are involved (e.g. Prevotella histicola seems elevated in both conditions). Meanwhile, the anxiety-specific marker Oribacterium did not appear for depression, hinting at a possible difference in microbial pattern for anxiety.

In the pregnant women cohort (Alex et al. 2024), anxiety symptoms were linked with higher abundance of certain taxa like *Dialister* and some Firmicutes. As mentioned, *Dialister* was higher in both high-anxiety and high-depression groups in that sample. Also, *Eikenella* – which can be considered a marker of periodontal disturbance – was elevated broadly in women with high anxiety, depression, or PTSD. This broad association could indicate that *Eikenella* thrives in any scenario of chronic stress or immune alteration. The pregnancy study emphasises that different mental health symptoms (stress, anxiety, depression, PTSD) each had their own microbial associations, with minimal overlap except for a few like *Eikenella*. It’s notable that anxiety (especially trait anxiety) was associated with higher Firmicutes and a more diverse microbiome (Alex et al. 2024). Firmicutes include many streptococci and lactobacilli; a shift towards more Firmicutes might mean changes in oral pH or diet (e.g. high sugar intake can boost Firmicutes like streptococci). Anxiety could plausibly lead to habits like increased sugar consumption (stress-eating) or dry mouth (from hyperventilation or medications), indirectly shaping the microbiome.

PTSD-related symptoms should be interpreted separately from anxiety disorders. In the present review, PTSD-related findings were included only because trauma-related symptoms overlap with anxiety and depression and because inflammatory pathways may be shared across stress-related conditions. However, consistent with DSM-5/DSM-5-TR, PTSD is a trauma- and stressor-related disorder, not an anxiety disorder. Therefore, summary tables and text refer to anxiety/PTSD-related findings only when the distinction is made explicit (Malan-Müller et al. 2024).

### Integrating inflammation and oral microbiome

An important point of convergence between these two domains is inflammation. Oral microbiome dysbiosis can lead to local including gingivitis and periodontitis, which may influence systemic inflammatory markers such as CRP and IL-6. It is therefore plausible that oral inflammation could contribute to the low-grade inflammatory burden observed in some individuals with depression. However, none of the included studies directly established mediation from oral bacteria to systemic inflammation to psychiatric symptoms, so this pathway remains hypothetical.

It is also important to consider behavioural and clinical confounders. Depression and anxiety can be accompanied by changes in oral hygiene practices, diet, smoking, alcohol use, and health care utilisation. In addition, antidepressants and anxiolytics may have anticholinergic effects that contribute to xerostomia, which can substantially alter salivary flow and oral microbial ecology. Dry mouth can favour acidogenic or anaerobic organisms and may interact with periodontal disease, dental plaque, or caries risk. These factors were inconsistently measured across studies and therefore limit causal interpretation of oral microbiome findings (Alex et al. 2024).

In conclusion, depression and anxiety are associated with measurable changes in the oral microbiome, but these findings are preliminary and heterogeneous. Depression often aligns with shifts towards taxa linked to oral dysbiosis or inflammation, although specific taxa vary by population and methodology. Anxiety and trauma-related symptoms may also influence the oral microbiome, perhaps through stress physiology, behaviour, medication effects, or oral-health pathways. The oral cavity may therefore be both a reflection of mental health-related behaviours and a potential contributor to systemic inflammatory burden, but causal claims require longitudinal and mechanistic confirmation.

## Discussion

**Figure 2.**
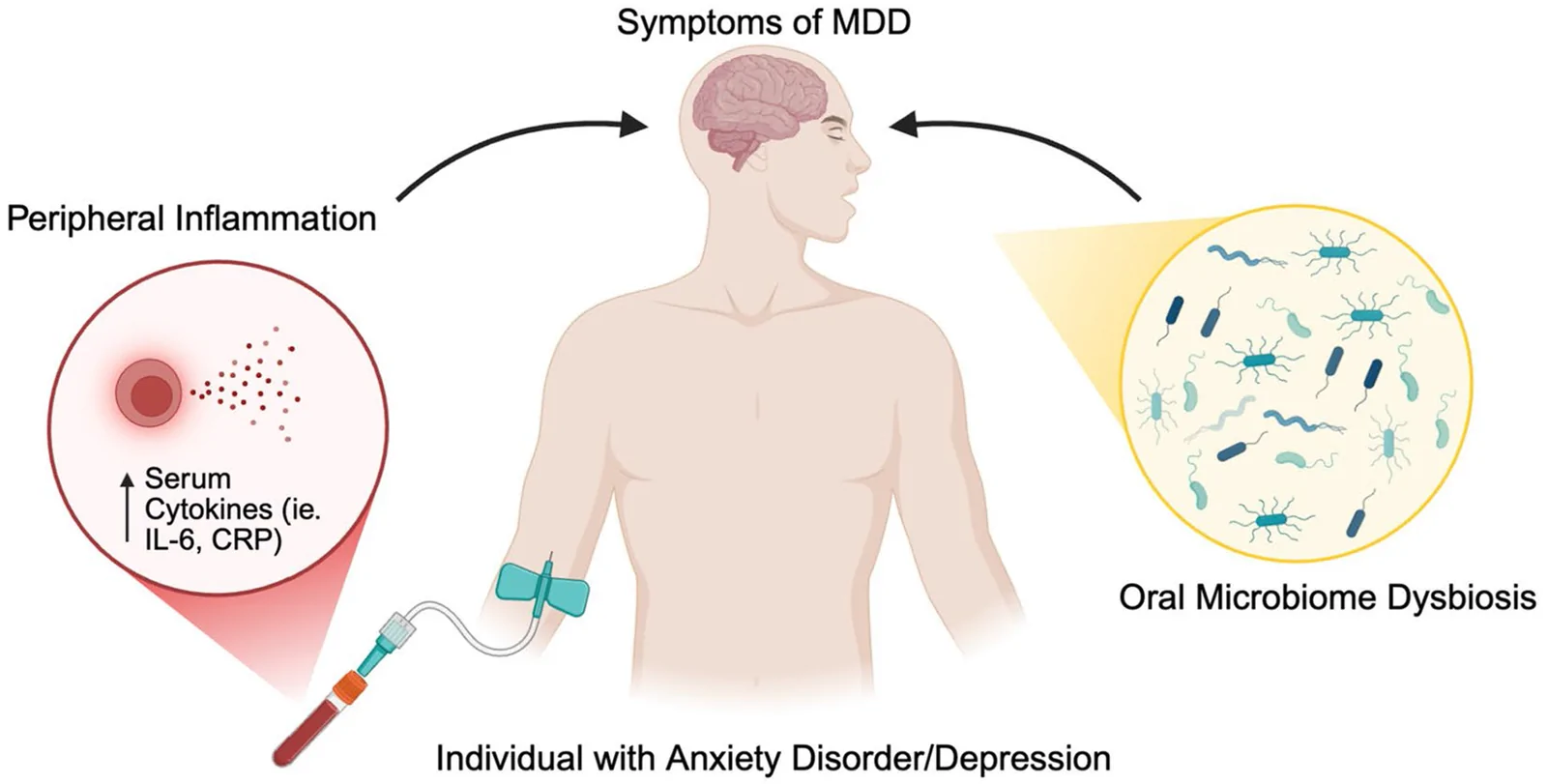
Conceptual model illustrating potential associations among peripheral inflammation, oral microbiome dysbiosis, and depression/anxiety-related symptoms. The diagram is intended as a conceptual framework rather than evidence of causality.

In this systematic review, we synthesised recent evidence linking depression and anxiety with peripheral inflammation and the oral microbiome, illustrated in Figure 2. The findings reinforce an increasingly nuanced view of the mind-body connection: depression, in particular, is associated with a pro-inflammatory state and concurrent alterations in oral microbial communities. Anxiety disorders, while sometimes overlapping with depression in these changes, show a more complex and sometimes weaker association.

### Interpreting the inflammation findings

The association between depression and elevated inflammatory markers such as CRP and IL-6 suggests that immune dysregulation is relevant for a subset of patients. However, the magnitude of inflammatory elevation in depression is typically modest. CRP has clinically interpretable reference ranges, whereas IL-6 and most cytokines do not have standardised psychiatric clinical cut-offs. Therefore, statistically significant differences in IL-6 or other cytokines should not automatically be interpreted as clinically meaningful. From a pathophysiological perspective, chronic low-grade inflammation may influence neurotransmitter metabolism, neuronal plasticity, and HPA-axis function, but these mechanisms remain inferential in most human studies.

It’s important to note that not all depressed individuals have elevated inflammation This likely reflects different “biotypes” or subgroups of depression. Some depressed patients, such as those with early-life stress, obesity, medical comorbidity, or somatic symptoms, may show stronger inflammatory signatures, whereas others may not. Symptom-specific associations linking inflammation more strongly with fatigue, sleep, appetite, and anhedonia are especially relevant but require further replication before clinical application.

For anxiety, the discussion is more nuanced. The lack of a uniform inflammatory signature for anxiety might indicate that different anxiety disorders need to be considered separately. GAD, characterised by chronic worry, might involve more HPA axis activation and cortisol release, which can suppress certain inflammatory pathways (potentially explaining findings like lower IL-1β in GAD) (Shen et al. 2022). PTSD, on the other hand, can involve periods of hyperarousal and hypoarousal, immune suppression followed by exaggerated inflammatory responses (some describe PTSD as having a “biphasic” immune profile). Our review suggests that when anxiety occurs alongside depression or severe stress, inflammation is often present, but in pure anxiety it may not be. This could explain why trials of anti-inflammatory agents in pure anxiety are virtually nonexistent – because there hasn’t been a clear signal that inflammation is a maintenance factor in those disorders. An implication for future research is to identify when during the course of anxiety disorders immune changes occur. It is possible that during acute anxiety episodes (e.g. panic attacks or acute stress) there are transient spikes in inflammation, but chronically anxious individuals might acclimate, leading to normal baseline levels. Longitudinal studies measuring cytokines repeatedly in anxious patients could clarify this dynamic.

### Interpreting the oral microbiome findings

The oral microbiome is a novel player in the landscape of depression/anxiety research, and our review suggests that the oral cavity may mirror broader health states. For instance, systemic diseases such as diabetes and other chronic inflammatory conditions can alter oral flora. Mental health conditions may similarly register in the oral microbiome through indirect pathways, including behavioural changes, stress physiology, salivary flow, and oral immune changes.

One interpretation is that depression and anxiety often involve behaviours that can modulate the oral microbiome: poor oral hygiene, altered diet, smoking, bruxism, mouth breathing, medication-related xerostomia, and reduced dental care. These behavioural and clinical factors are not merely limitations; they may be mediators or confounders of the observed oral microbiome differences. For example, depressed individuals with increased Prevotella and Fusobacterium may also have greater plaque accumulation or periodontal inflammation. Without detailed oral-health metadata, it is difficult to determine whether microbial differences reflect psychiatric status, oral disease, medication effects, or a combination of these pathways.

However, we should not overly attribute the changes solely to hygiene. The pregnancy study is telling: all women were presumably receiving prenatal care and likely had at least moderate health behaviours, yet significant differences in microbes were found purely based on stress and mood levels (Alex et al. 2024). This suggests physiological stress pathways (like cortisol, immunoglobulin A in saliva, or adrenergic signalling in gums) might directly alter microbial composition. Stress is known to change the mouth’s environment – for instance, high cortisol can reduce IgA and increase susceptibility to opportunistic bacteria; sympathetic activation can alter blood flow to gums. Over time, these physiologic changes might select for certain bacteria that handle stress-related conditions better (like those that thrive in a more inflammatory milieu or drier mouth). The finding that *Dialister* was higher in high-anxiety/depression pregnant women could reflect such a process; *Dialister* is often anaerobic and might take advantage of niches created by changes in salivary flow or oxygen.

Another piece of the puzzle is the functional metabolic output of the microbiome. The involvement of tryptophan-related pathways is intriguing but should be treated cautiously. Current evidence does not demonstrate that oral bacteria consume enough tryptophan during mastication or deglutition to meaningfully alter systemic serotonin availability. Thus, predicted functional pathways should be interpreted as exploratory biomarkers or hypotheses rather than direct mechanistic proof. Stronger mechanistic studies are needed before claims about oral microbial effects on serotonin can be made.

In practical terms, the oral microbiome findings hint at potential biomarkers or intervention targets. Saliva samples are easy to collect – if specific microbial patterns (like high *Prevotella*/low *Neisseria*) were validated as correlating with depression severity or treatment resistance, they could be used to identify high-risk individuals or monitor treatment response. Interventions like improving oral hygiene or treating periodontal disease might also reduce inflammatory burden, but whether such interventions improve psychiatric symptoms remains unknown. Given the accessibility of the oral environment, future trials that integrate dental care, inflammatory biomarkers, and psychiatric outcomes are warranted.

### Oral microbiome compared with gut microbiome

Although this review focuses on the oral microbiome, the findings should be contextualised within the broader gut microbiome-gut-brain axis literature. The gut microbiome has been studied more extensively in depression and anxiety, and gut-derived metabolites, immune pathways, endocrine signalling, and vagal mechanisms are more developed mechanistically. In contrast, the oral microbiome is more directly shaped by local factors such as salivary flow, plaque, periodontal disease, oral hygiene, diet, smoking, and medication-related xerostomia. Both microbial niches may intersect with systemic inflammation and mental health, but oral microbiome findings should not be assumed to operate through the same mechanisms as gut microbiome findings. Rather, the oral cavity may provide a complementary and accessible biomarker niche within a broader microbiome-immune-brain framework (Patel et al. 2025).

### Limitations

This review has several limitations. First, although we performed an updated systematic search across PubMed, Web of Science, and PsycINFO from 2016 to 2026, relevant studies may still have been missed because terminology varies across psychiatry, immunology, and microbiome research. Second, the heterogeneity of psychiatric definitions, biomarker assays, sampling methods, and covariate adjustment precluded meta-analysis. We therefore used qualitative synthesis and emphasised consistency, study design, and risk of bias. Third, although risk of bias was assessed using NOS/adapted NOS criteria, study quality remains limited by small samples, cross-sectional designs, and incomplete confounder adjustment in several studies.

Many studies were cross-sectional, limiting causal inference. We often don’t know whether inflammation or microbiome changes are a cause or a consequence of depression/anxiety. The longitudinal studies begin to address temporality, but they remain observational. Unmeasured confounders also complicate interpretation. For inflammation, BMI, diet, medical illnesses, infection, and medication use can influence biomarker levels. For the oral microbiome, oral hygiene, periodontal disease, caries, xerostomia, antibiotics, smoking, diet, and socioeconomic status are especially important.

Additionally, the generalisability of these findings may be limited by the demographic and clinical makeup of samples. Many studies were from high-income countries and some focused on specific groups (e.g. mostly women in the prenatal study, relatively young adults in others). The oral microbiome varies with age and diet (for instance, adolescents often have different oral bacteria than older adults). Depression in a 20-year-old student might manifest differently in terms of microbiome than depression in a 70-year-old retired person. None of the included microbiome studies looked at older adults or very long-term chronically depressed individuals, so that remains an open area.

For the oral microbiome results specifically, a limitation is that most studies used 16S rRNA gene sequencing which provides genus-level (and some species-level) identification but not functional data. Different studies targeted different regions of the 16S gene and used different bioinformatic pipelines, which can lead to some differences in taxonomic resolution. This makes direct comparison of microbial lists across studies tricky. Moreover, the microbiome is inherently complex – factors like oral pH, saliva flow rate, and oral immune components weren’t measured in these studies but would help interpret why certain microbes proliferate. We also note that “oral microbiome” in these studies refers to saliva microbiome; there are distinct niches (tongue, plaque, tonsillar crypts) that could have different relationships with systemic conditions. Saliva is a mix of all niches and might dilute some niche-specific signals.

## Future directions

The intersection of mental health with immunology and microbiology opens several future research avenues. Longitudinal and interventional studies are critical. For inflammation, future trials should stratify participants by baseline inflammatory burden and distinguish MDD from broader depressive symptomatology. For anxiety, immune studies should separate GAD, panic, PTSD-related symptoms, and anxiety comorbid with depression. It would also be informative to examine whether successful psychiatric treatment normalises inflammatory markers and whether changes differ by symptom domain.

Regarding the oral microbiome, future studies should strive for larger sample sizes and longitudinal designs. Does the oral microbiome change as people develop depression or does it change as people recover? Answering these questions requires repeated psychiatric assessment, concurrent oral microbiome sampling, systemic and salivary inflammatory markers, and detailed oral-health metadata. Future studies should also measure psychotropic medication use, xerostomia, smoking, periodontal status, oral hygiene behaviours, antibiotic exposure, diet, and dental care access.

Mechanistic studies are also needed. Animal and experimental models could help generate hypotheses, but human longitudinal and interventional studies will be essential for clinical translation. Studies that jointly assess periodontal inflammation, salivary flow, microbial taxa, microbial function, circulating inflammatory markers, and psychiatric symptoms would be especially informative.

From a clinical standpoint, our review highlights the importance of integrative care. Patients with depression and anxiety often see primary care for somatic complaints; knowing that inflammation might be part of their illness could prompt screening for inflammatory risks (like metabolic syndrome) and holistic management. Likewise, mental health professionals should be aware that their patients might have unmet general health needs – for example, a depressed patient with severe dental problems might benefit from a coordinated referral to dentistry, which could indirectly improve their psychiatric condition (through reduction of pain, improvement of self-image, and possibly lowering inflammatory load). In essence, treating the whole person includes attending to physical health and lifestyle, which in turn can help mental well-being.

## Conclusion

Depression and anxiety are complex disorders with multifactorial aetiologies. The evidence reviewed here underscores that psychological distress is not isolated from the body’s physiology. In depressed individuals, elevated acute-phase proteins such as CRP and cytokine-related changes such as IL-6 signalling point to chronic, low-grade inflammatory activity in a subset of patients. Anxiety disorders show a less uniform immune response, but when anxiety is coupled with depression, stress, or trauma-related symptoms, inflammatory activation often emerges as part of the clinical picture. These insights reinforce the idea that biomarkers may eventually help guide more personalised assessment and treatment, while also highlighting the need for cautious interpretation.

Concurrently, the oral microbiome may play a role in the psychobiology of depression and anxiety-related symptoms. The oral cavity’s microbial community appears sensitive to mood, stress, and anxiety-related symptoms, with shifts that may reflect local oral health, systemic inflammatory signalling, salivary flow, medication effects, and behavioural changes. While it is too early to draw firm clinical implications, salivabased microbial and inflammatory measures may eventually serve as accessible indicators of chronic stress or depression risk if validated in larger longitudinal studies.

In moving forward, a more integrated approach to research and treatment is warranted – one that bridges psychiatry with immunology and oral biology. Collaborations between mental health professionals, immunologists, and dental/oral health researchers will be valuable for clarifying mechanisms and developing interventions that address both physical and mental health. Ultimately, such interdisciplinary efforts may improve understanding of depression and anxiety and support more holistic approaches to care.

## Supplementary Material

Supp 1

Supplemental data for this article can be accessed online at https://doi.org/10.1080/15622975.2026.2688864.

## Figures and Tables

**Table 1.** Key inflammatory and salivary biomarkers associated with depression and anxiety-related outcomes (2016–2026 human studies).

Biomarker/domain	Depression/depressive symptoms	Anxiety/GAD/PTSD-related symptoms	Interpretation and representative evidence
CRP / hsCRP	Frequently associated with depressive symptom burden, recurrent or severe depression, and somatic symptom dimensions in several primary studies.	CRP-anxiety associations were less consistent; population studies suggest small associations that may depend on comorbid depression and medical covariates.	CRP has clinically interpretable ranges, but psychiatric associations are usually modest. Representative studies include Naudé et al.; Kappelmann et al.; Zainal & Newman; Shi et al.; Brunoni et al.
IL-6 / IL-6-related signalling	Often elevated or associated with depression, anhedonia, cognition, or symptom severity, but absolute differences may be small.	Mixed findings in GAD and anxiety; some studies report lower or null IL-6 associations.	No universally accepted psychiatric clinical cut-off exists for IL-6. Interpret small mean differences cautiously. Representative studies include Nishuty et al.; Foley et al.; Ye et al.; Wagner et al.
TNF-α, IL-1β, IL-18, NLRP3	Several studies report higher TNF-α, IL-1β, IL-18, or inflammasome-related markers in MDD or medication-naïve depression samples.	Sparse and inconsistent evidence in anxiety-specific samples.	These markers support immune dysregulation in some depressive subgroups but are not diagnostic biomarkers.
Anti-inflammatory cytokines (IL-4, IL-10) and cytokine balance	Some studies report altered anti-inflammatory cytokines or pro-/anti-inflammatory balance in MDD.	GAD studies report variable patterns, including possible compensatory anti-inflammatory changes.	Direction and meaning depend on population, medication status, and assay methods.
Chemokines, complement, SOCS, inflammatory ratios	Emerging studies report CC chemokines, complement-related markers, suppressors of cytokine signalling, and blood-cell ratios as candidate biomarkers.	Limited anxiety-specific evidence.	Promising but heterogeneous; most require replication and external validation.
Salivary inflammatory biomarkers	Altered salivary cytokines have been reported in MDD, but evidence is limited.	Salivary CRP/cytokines have been linked to trauma-related symptom severity in prospective work.	Saliva is accessible but strongly influenced by local oral inflammation, smoking, xerostomia, and periodontal disease.

Note. GAD = generalised anxiety disorder; PTSD is considered trauma- and stressor-related in DSM-5/DSM-5-TR and is listed separately when relevant. IL-6 and most cytokines do not have standardised psychiatric clinical cut-offs; CRP is more clinically interpretable.

**Table 2.** Summary of oral microbiome findings associated with depression and anxiety-related symptoms (2016–2026 human studies).

Microbiome feature/taxa	Depression/depressive symptoms	Anxiety/trauma-related symptoms	Interpretation and representative evidence
Overall diversity and community composition	Several studies report altered oral microbiome composition in depression or depressive symptoms; diversity findings vary by population.	Large community and symptom-based studies suggest altered composition with anxiety or trauma-related symptoms, but evidence is smaller.	Composition may be more informative than alpha diversity alone. Representative studies include Wingfield et al.; Simpson et al.; Malan-Müller et al.; Zheng et al.; Qiu et al.
*Neisseria*	Direction varies across studies: lower abundance in some depressive-symptom samples but higher or differentially abundant in others.	Lower Neisseria elongata has been reported in anxiety-related analyses.	Conflicting direction suggests strong influence of population, oral health, sample site, and sequencing approach.
*Prevotella, Fusobacterium, Porphyromonas*	Often interpreted as taxa with periodontal or inflammatory relevance when enriched in depression-associated profiles.	May appear in trauma-related or anxiety-associated profiles but not consistently.	These taxa should be treated as candidate dysbiosis markers, not causal agents. Periodontal disease is a key confounder.
*Streptococcus and Haemophilus*	Reported as differentially abundant or predictive in some adolescent and population studies.	Less consistent anxiety-specific signal.	These are common oral taxa; interpretation requires attention to species-level resolution and oral site.
Functional pathway predictions	Some studies suggest associations with microbial functional pathways, including tryptophan-related pathways.	Functional findings remain sparse.	Functional predictions are hypothesis-generating and should not be interpreted as evidence that oral bacteria causally reduce host serotonin.
Oral-health and behavioural confounders	Medication-related xerostomia, smoking, oral hygiene, diet, periodontal disease, antibiotics, and caries may alter microbial composition.	Same confounders may be especially important in anxiety or trauma-related samples due to smoking, stress physiology, and medication exposure.	Future studies should measure and adjust for oral-health metadata systematically.

**Note.** Higher or lower relative abundance refers to differences reported in psychiatric or higher-symptom groups compared with controls or lower-symptom groups. Findings are preliminary and should be interpreted as associations.

## Data Availability

The data supporting the findings of this study are derived from previously published studies and are available within the cited literature.

## KEY MESSAGES

1. Depression is associated with alterations in peripheral inflammatory biomarkers, particularly CRP and IL-6-related signalling, but many cytokine differences are modest and not necessarily clinically meaningful.
2. Emerging evidence indicates that depression and anxiety-related symptoms are associated with oral microbiome dysbiosis characterised by shifts in inflammatory and commensal bacterial taxa, although findings remain heterogeneous.
3. The oral microbiome may represent a novel component of an oral-immune-brain framework with potential implications for biomarker development and integrated dental-mental health care, but causal mechanisms remain unproven.
