# Supplementary material for: Inflammatory biomarkers and oral microbiome alterations in depression and anxiety disorders: a systematic review
Source: World J Biol Psychiatry. Author manuscript; Available in PMC 2026 Aug 7. (PMC13442454; doi:10.1080/15622975.2026.2688864)
Supplement: Supplementary file 1 [file NIHMS2197366-supplement-Supp_1.docx]

**Supplementary Material**

*Inflammatory Biomarkers and Oral Microbiome Alterations in Depression and Anxiety Disorders: A Systematic Review*

This supplementary file provides the full search strategy, risk-of-bias scoring rubric and quality assessment table, included-study list, and PRISMA support tables referenced in the manuscript.

# Supplementary Table S1. Full database search strategy.

Searches were conducted in PubMed, Web of Science, and PsycINFO using database-specific keyword queries. The table reports the exact search terms entered, search field, filters/date range, number of records retrieved, and search link for each query.

| **Search ID** | **Database** | **Search focus** | **Search field** | **Exact search terms entered** | **Filters/date range** | **Records retrieved (n)** | **Link** |
| --- | --- | --- | --- | --- | --- | --- | --- |
| 1 | Web of Science | Anxiety/GAD + inflammatory biomarkers | Topic | TS=(inflammatory biomarkers anxiety generalized anxiety disorder) | English; human; Jan 1 2016-Jan 1 2026 | 71 | <https://www.webofscience.com/wos/woscc/summary/30bc25d9-5ba4-41c9-b84f-f60bcc42822c-01b29ce0ea/78c47d3c-6fd7-44ef-bab5-b3af4a599862-01b29cdfa9/relevance/1> |
| 2 | Web of Science | Anxiety/GAD + salivary biomarkers | Topic | TS=(salivary biomarkers anxiety generalized anxiety disorder) | English; human; Jan 1 2016-Jan 1 2026 | 24 | <https://www.webofscience.com/wos/woscc/summary/264da8dc-ee68-4c3e-82e2-8f3ada7abd04-01b29bee4d/c14ef147-415f-4737-aac8-3259340978d5-01b29be91b/relevance/1> |
| 3 | Web of Science | Depression/MDD + inflammatory biomarkers | Topic | TS=(inflammatory biomarkers depression major depressive disorder) | English; human; Jan 1 2016-Jan 1 2026 | 376 | <https://www.webofscience.com/wos/woscc/summary/28a2d6f1-0580-4cb7-8a68-31bcea12c476-01b29d2952/a618e211-4534-4747-8e44-dcc04c7aad3c-01b29d284c/relevance/1> |
| 4 | Web of Science | Depression/MDD + salivary biomarkers | Topic | TS=(salivary biomarkers depression major depressive disorder) | English; human; Jan 1 2016-Jan 1 2026 | 36 | <https://www.webofscience.com/wos/woscc/summary/94368198-f6be-417d-a2b0-29bdac923b69-01b29bd9bc/9ed8f0a1-8f8b-4ed4-bcb4-03539d7c8c17-01b29bd7cf/relevance/1> |
| 5 | Web of Science | Anxiety/GAD + oral microbiome | Topic | (TS=(oral microbiome anxiety generalized anxiety disorder) | English; human; Jan 1 2016-Jan 1 2026 | 57 | <https://www.webofscience.com/wos/woscc/summary/bae6371b-646f-46d7-be11-ca8d28d25141-01b29cbdcc/fe5db0c8-6993-4136-ae70-035425139c59-01b29cbb6c/relevance/1> |
| 6 | Web of Science | Depression/MDD + oral microbiome | Topic | TS=(oral microbiome depression major depressive disorder) | English; human; Jan 1 2016-Jan 1 2026 | 70 | <https://www.webofscience.com/wos/woscc/summary/38dc0518-a24e-40da-98f3-ff8d40f8f78c-01b29cc126/2c52453e-3a1f-49d8-b5a8-248b36055a7c-01b29cbfc5/relevance/1> |
| 7 | PsycINFO | Anxiety/GAD + salivary biomarkers | All fields | (salivary biomarkers anxiety generalized anxiety disorder) | English; peer-reviewed/human where available; Jan 1 2016-Jan 1 2026 | 6 | <https://www.proquest.com/psycinfo/results/FC7419C1DB8B4824PQ/1?accountid=14512> |
| 8 | PsycINFO | Anxiety/GAD + inflammatory biomarkers | All fields | (inflammatory biomarkers anxiety generalized anxiety disorder) | English; peer-reviewed/human where available; Jan 1 2016-Jan 1 2026 | 30 | <https://www.proquest.com/psycinfo/results/83C07E421B8A4759PQ/1?accountid=14512> |
| 9 | PsycINFO | Depression/MDD + salivary biomarkers | All fields | (salivary biomarkers depression major depressive disorder) | English; peer-reviewed/human where available; Jan 1 2016-Jan 1 2026 | 22 | <https://www.proquest.com/psycinfo/results/12AB721E864F4380PQ/1?accountid=14512> |
| 10 | PsycINFO | Depression/MDD + inflammatory biomarkers | All fields | (inflammatory biomarkers depression major depressive disorder) | English; peer-reviewed/human where available; Jan 1 2016-Jan 1 2026 | 402 | <https://www.proquest.com/psycinfo/results/AE99592B85004F71PQ/1?accountid=14512> |
| 11 | PsycINFO | Anxiety/GAD + oral microbiome | All fields | (oral microbiome anxiety generalized anxiety disorder) | English; peer-reviewed/human where available; Jan 1 2016-Jan 1 2026 | 1 | <https://www.proquest.com/psycinfo/results/C89FD4D7CE804C08PQ/1?accountid=14512> |
| 12 | PsycINFO | Depression/MDD + oral microbiome | All fields | (oral microbiome depression major depressive disorder) | English; peer-reviewed/human where available; Jan 1 2016-Jan 1 2026 | 8 | <https://www.proquest.com/psycinfo/results/50C925F826C947E6PQ/1?accountid=14512> |
| 13 | PubMed | Anxiety/GAD + salivary biomarkers | Title/Abstract | (salivary biomarkers anxiety generalized anxiety disorder) | English; Jan 1 2016-Jan 1 2026 | 7 | <https://pubmed.ncbi.nlm.nih.gov/?term=salivary+biomarkers+anxiety+generalized+anxiety+disorder&filter=dates.2016%2F1%2F1-2026%2F1%2F1&size=100> |
| 14 | PubMed | Anxiety/GAD + inflammatory biomarkers | Title/Abstract | (inflammatory biomarkers anxiety generalized anxiety disorder) | English; Jan 1 2016-Jan 1 2026 | 35 | <https://pubmed.ncbi.nlm.nih.gov/?term=inflammatory+biomarkers+anxiety+generalized+anxiety+disorder&filter=dates.2016%2F1%2F1-2026%2F1%2F1&size=100> |
| 15 | PubMed | Depression/MDD + salivary biomarkers | Title/Abstract | (salivary biomarkers depression major depressive disorder) | English; Jan 1 2016-Jan 1 2026 | 21 | <https://pubmed.ncbi.nlm.nih.gov/?term=salivary+biomarkers+depression+major+depressive+disorder&filter=dates.2016%2F1%2F1-2026%2F1%2F1&size=100> |
| 16 | PubMed | Depression/MDD + inflammatory biomarkers | Title/Abstract | (inflammatory biomarkers depression major depressive disorder) | English; Jan 1 2016-Jan 1 2026 | 650 | <https://pubmed.ncbi.nlm.nih.gov/?term=inflammatory+biomarkers+depression+major+depressive+disorder&filter=dates.2016%2F1%2F1-2026%2F1%2F1&size=100> |
| 17 | PubMed | Anxiety/GAD + oral microbiome | Title/Abstract | (oral microbiome anxiety generalized anxiety disorder) | English; Jan 1 2016-Jan 1 2026 | 1 | <https://pubmed.ncbi.nlm.nih.gov/36389063/> |
| 18 | PubMed | Depression/MDD + oral microbiome | Title/Abstract | (oral microbiome depression major depressive disorder) | English; Jan 1 2016-Jan 1 2026 | 31 | <https://pubmed.ncbi.nlm.nih.gov/?term=oral+microbiome+depression+major+depressive+disorder&size=100&filter=dates.2016%2F1%2F1-2026%2F1%2F1> |

# Supplementary Table S2. Risk-of-bias and quality assessment using the Newcastle–Ottawa Scale/adapted Newcastle–Ottawa framework.

NOS/adapted NOS scoring rubric: Risk of bias was assessed using the Newcastle–Ottawa Scale (NOS) for cohort and case-control studies and an adapted NOS framework for cross-sectional studies. Studies were scored across three domains: selection (0–4 points), comparability (0–2 points), and outcome/exposure assessment (0–3 points), for a maximum score of 9. Selection considered clarity and representativeness of the study population, appropriateness of comparison groups, psychiatric assessment method, and biological exposure/outcome measurement. Comparability considered adjustment for demographic and clinical confounders, including age, sex, BMI, smoking status, medication use, periodontal disease, oral hygiene behaviors, and antibiotic exposure where relevant. Outcome/exposure assessment considered validity of biomarker or microbiome methods, validity of psychiatric outcome measurement, and appropriateness of statistical analysis. Total scores of 7–9 were classified as low risk of bias, 4–6 as moderate risk of bias, and 0–3 as high risk of bias.

| **Study ID** | **Title** | **Evidence track** | **Source** | **Study design** | **Selection (0-4)** | **Comparability (0-2)** | **Outcome/exposure (0-3)** | **Total score** | **Risk of bias** | **Notes** |
| --- | --- | --- | --- | --- | --- | --- | --- | --- | --- | --- |
| S1 | Association between oral microbiome and depression: A population-based study | Oral microbiome | Database search | Population-based cross-sectional / secondary data analysis | 4 | 2 | 3 | 9 | Low | Quality assessment based on extracted study characteristics and reported methods. |
| S2 | Associations Between Oral Microbiota Pathogens and Elevated Depressive and Anxiety Symptoms in Men | Oral microbiome | Database search | Cross-sectional observational | 4 | 2 | 3 | 9 | Low | Quality assessment based on extracted study characteristics and reported methods. |
| S3 | Oral microbiome composition, but not diversity, is associated with adolescent anxiety and depression symptoms | Oral microbiome | Database search | Cross-sectional observational | 4 | 1 | 3 | 8 | Low | Quality assessment based on extracted study characteristics and reported methods. |
| S4 | Probing the oral-brain connection: oral microbiome patterns in a large community cohort with anxiety, depression, and trauma symptoms, and periodontal outcomes | Oral microbiome | Database search | Large cross-sectional community cohort | 4 | 2 | 3 | 9 | Low | Quality assessment based on extracted study characteristics and reported methods. |
| S5 | Relationship between depression and oral microbiome diversity: analysis of NHANES data (2009-2012) | Oral microbiome | Database search | Population-based cross-sectional / NHANES analysis | 4 | 2 | 3 | 9 | Low | Quality assessment based on extracted study characteristics and reported methods. |
| S6 | The association of depression and oral microbiota | Oral microbiome | Database search | Cross-sectional observational | 4 | 1 | 3 | 8 | Low | Quality assessment based on extracted study characteristics and reported methods. |
| S7 | Variations in the oral microbiome are associated with depression in young adults | Oral microbiome | Database search | Case-control observational | 4 | 1 | 3 | 8 | Low | Quality assessment based on extracted study characteristics and reported methods. |
| S8 | Altered levels of salivary cytokines in patients with major depressive disorder | Salivary inflammatory biomarkers | Database search | Case-control observational | 4 | 1 | 3 | 8 | Low | Quality assessment based on extracted study characteristics and reported methods. |
| S9 | Altered Serum IL-6 and TGF-β1 Levels Are Associated With Generalized Anxiety Disorder: A Case-Control Study | Systemic inflammatory biomarkers - anxiety | Database search | Case-control observational | 4 | 1 | 3 | 8 | Low | Quality assessment based on extracted study characteristics and reported methods. |
| S10 | Altered serum interleukin-17A and interleukin-23A levels may be associated with the pathophysiology and development of generalized anxiety disorder | Systemic inflammatory biomarkers - anxiety | Database search | Case-control observational | 4 | 1 | 3 | 8 | Low | Quality assessment based on extracted study characteristics and reported methods. |
| S11 | Anxiety disorders and CRP in a population cohort study with 54,326 participants: The LifeLines study | Systemic inflammatory biomarkers - anxiety | Database search | Population cohort / cross-sectional analysis | 4 | 2 | 3 | 9 | Low | Quality assessment based on extracted study characteristics and reported methods. |
| S12 | Association of interleukin-2 and interleukin-10 with the pathophysiology and development of generalized anxiety disorder: a case-control study | Systemic inflammatory biomarkers - anxiety | Database search | Case-control observational | 4 | 1 | 3 | 8 | Low | Quality assessment based on extracted study characteristics and reported methods. |
| S13 | Generalized Anxiety Disorder is Prospectively Associated With Decreased Levels of Interleukin-6 and Adiponectin Among Individuals from the Community | Systemic inflammatory biomarkers - anxiety | Database search | Prospective community cohort | 4 | 2 | 3 | 9 | Low | Quality assessment based on extracted study characteristics and reported methods. |
| S14 | A novel biomarker of interleukin 6 activity and clinical and cognitive outcomes in depression | Systemic inflammatory biomarkers - depression | Database search | Observational biomarker study | 4 | 1 | 3 | 8 | Low | Quality assessment based on extracted study characteristics and reported methods. |
| S15 | Altered serum TNF-α and MCP-4 levels are associated with the pathophysiology of major depressive disorder: A case-control study results | Systemic inflammatory biomarkers - depression | Database search | Case-control observational | 4 | 1 | 3 | 8 | Low | Quality assessment based on extracted study characteristics and reported methods. |
| S16 | Altered serum interleukin-7 and interleukin-10 are associated with drug-free major depressive disorder | Systemic inflammatory biomarkers - depression | Database search | Case-control observational | 4 | 1 | 3 | 8 | Low | Quality assessment based on extracted study characteristics and reported methods. |
| S17 | Altered serum levels of TNF-α, IL-6, and IL-18 in depressive disorder patients | Systemic inflammatory biomarkers - depression | Database search | Case-control observational | 4 | 1 | 3 | 8 | Low | Quality assessment based on extracted study characteristics and reported methods. |
| S18 | Association between C-reactive protein (CRP) with depression symptom severity and specific depressive symptoms in major depression | Systemic inflammatory biomarkers - depression | Database search | Cross-sectional observational / symptom analysis | 4 | 2 | 3 | 9 | Low | Quality assessment based on extracted study characteristics and reported methods. |
| S19 | Association between inflammatory cytokines and symptoms of major depressive disorder in adults | Systemic inflammatory biomarkers - depression | Database search | Cross-sectional observational | 4 | 1 | 3 | 8 | Low | Quality assessment based on extracted study characteristics and reported methods. |
| S20 | Associations of number and severity of depressive episodes with C-reactive protein and Interleukin-6 | Systemic inflammatory biomarkers - depression | Database search | Observational clinical study | 4 | 2 | 3 | 9 | Low | Quality assessment based on extracted study characteristics and reported methods. |
| S21 | Cytokine-Related Genes and Inflammatory Profiles as Potential Biomarkers in Major Depressive Disorder | Systemic inflammatory biomarkers - depression | Database search | Case-control / biomarker profile study | 4 | 1 | 3 | 8 | Low | Quality assessment based on extracted study characteristics and reported methods. |
| S22 | Evaluation of Serum Interleukin-6 and C-reactive Protein Levels in Drug-naive Major Depressive Disorder Patients | Systemic inflammatory biomarkers - depression | Database search | Case-control observational | 4 | 1 | 3 | 8 | Low | Quality assessment based on extracted study characteristics and reported methods. |
| S23 | Evaluation of serum interleukin-1 receptor antagonist levels in major depressive disorder: A case-control study | Systemic inflammatory biomarkers - depression | Database search | Case-control observational | 4 | 1 | 3 | 8 | Low | Quality assessment based on extracted study characteristics and reported methods. |
| S24 | Evaluation of serum interleukin-12 and interleukin-4 as potential biomarkers for the diagnosis of major depressive disorder | Systemic inflammatory biomarkers - depression | Database search | Case-control observational | 4 | 1 | 3 | 8 | Low | Quality assessment based on extracted study characteristics and reported methods. |
| S25 | Faster speed of onset of the depressive episode is associated with lower cytokine serum levels (IL-2,-4,-6,-10, TNF-α and IFN-γ) in patients with major depression | Systemic inflammatory biomarkers - depression | Database search | Cross-sectional clinical observational | 3 | 1 | 3 | 7 | Low | Quality assessment based on extracted study characteristics and reported methods. |
| S26 | Higher levels of serum IL-1β and TNF-α are associated with an increased probability of major depressive disorder | Systemic inflammatory biomarkers - depression | Database search | Case-control observational | 4 | 1 | 3 | 8 | Low | Quality assessment based on extracted study characteristics and reported methods. |
| S27 | Higher serum interleukin-12 levels are associated with the pathophysiology of major depressive disorder: A case-control study results | Systemic inflammatory biomarkers - depression | Database search | Case-control observational | 4 | 1 | 3 | 8 | Low | Quality assessment based on extracted study characteristics and reported methods. |
| S28 | Inflammatory cytokines, complement factor H and anhedonia in drug-naïve major depressive disorder | Systemic inflammatory biomarkers - depression | Database search | Case-control observational | 4 | 1 | 3 | 8 | Low | Quality assessment based on extracted study characteristics and reported methods. |
| S29 | Pro- and anti-inflammatory cytokines, but not CRP, are inversely correlated with severity and symptoms of major depression | Systemic inflammatory biomarkers - depression | Database search | Cross-sectional clinical observational | 4 | 1 | 3 | 8 | Low | Quality assessment based on extracted study characteristics and reported methods. |
| S30 | Prospective associations between hsCRP and GlycA inflammatory biomarkers and depression: The Brazilian longitudinal study of adult health (ELSA-Brasil) | Systemic inflammatory biomarkers - depression | Database search | Prospective cohort | 4 | 2 | 3 | 9 | Low | Quality assessment based on extracted study characteristics and reported methods. |
| S31 | Serum CC Chemokines as Potential Biomarkers for the Diagnosis of Major Depressive Disorder | Systemic inflammatory biomarkers - depression | Database search | Case-control biomarker study | 4 | 1 | 3 | 8 | Low | Quality assessment based on extracted study characteristics and reported methods. |
| S32 | Serum FAM19A5 levels: A novel biomarker for neuroinflammation and neurodegeneration in major depressive disorder | Systemic inflammatory biomarkers - depression | Database search | Case-control observational | 4 | 1 | 3 | 8 | Low | Quality assessment based on extracted study characteristics and reported methods. |
| S33 | Serum levels of interleukin-33 and mesencephalic astrocyte derived neurotrophic factors in patients with major depressive disorder: a cross-sectional comparative design | Systemic inflammatory biomarkers - depression | Database search | Cross-sectional comparative | 4 | 1 | 3 | 8 | Low | Quality assessment based on extracted study characteristics and reported methods. |
| S34 | Suppressors of Cytokine Signaling Are Decreased in Major Depressive Disorder Patients | Systemic inflammatory biomarkers - depression | Database search | Case-control molecular/inflammatory signaling study | 4 | 1 | 3 | 8 | Low | Quality assessment based on extracted study characteristics and reported methods. |
| S35 | The relationship between serum resolvin D1, NLRP3, cytokine levels, and adolescents with first-episode medication-naïve major depressive disorder | Systemic inflammatory biomarkers - depression | Database search | Case-control observational | 4 | 1 | 3 | 8 | Low | Quality assessment based on extracted study characteristics and reported methods. |
| S36 | The relationship of peripheral blood cell inflammatory biomarkers and psychological stress in unmedicated major depressive disorder | Systemic inflammatory biomarkers - depression | Database search | Cross-sectional observational | 4 | 1 | 3 | 8 | Low | Quality assessment based on extracted study characteristics and reported methods. |
| S37 | Youth depression and inflammation: Cross-sectional network analyses of C-Reactive protein, interleukin-6 and symptoms in a population-based sample | Systemic inflammatory biomarkers - depression | Database search | Population-based cross-sectional network analysis | 4 | 2 | 3 | 9 | Low | Quality assessment based on extracted study characteristics and reported methods. |
| S38 | Cytokine profile in first-episode drug-naive major depressive disorder patients with or without anxiety | Systemic inflammatory biomarkers - depression/anxiety | Database search | Case-control observational | 4 | 1 | 3 | 8 | Low | Quality assessment based on extracted study characteristics and reported methods. |
| S39 | Inflammation, depression, and anxiety disorder: A population-based study examining the association between Interleukin-6 and the experiencing of depressive and anxiety symptoms | Systemic inflammatory biomarkers - depression/anxiety | Database search | Population-based observational | 4 | 2 | 3 | 9 | Low | Quality assessment based on extracted study characteristics and reported methods. |
| S40 | New Insights into the oral microbiota and host epigenetic changes in obsessive compulsive disorder and major depressive disorder: Focus on BDNF | Oral microbiome | Database search | Case-control / comparative observational | 3 | 1 | 3 | 7 | Low | Quality assessment based on extracted study characteristics and reported methods. |
| S41 | HIGH-SENSITIVITY C-REACTIVE PROTEIN, POSSIBLE BIOMARKER FOR DEPRESSION IN ELDERLY POPULATION | Systemic inflammatory biomarkers - depression | Database search | Cross-sectional observational | 3 | 1 | 2 | 6 | Moderate | Quality assessment based on extracted study characteristics and reported methods. |
| S42 | High levels of TNF-α are associated with symptoms of depression in health professionals at a hospital | Systemic inflammatory biomarkers - depression | Database search | Cross-sectional occupational sample | 3 | 1 | 2 | 6 | Moderate | Quality assessment based on extracted study characteristics and reported methods. |
| S43 | Serum cytokines-based biomarkers in the diagnosis and monitoring of therapeutic response in patients with major depressive disorder | Systemic inflammatory biomarkers - depression | Database search | Observational clinical / treatment-monitoring component | 4 | 1 | 3 | 8 | Low | Quality assessment based on extracted study characteristics and reported methods. |
| S44 | Role of inflammation in depression and anxiety: Tests for disorder specificity, linearity and potential causality of association in the UK Biobank | Systemic inflammatory biomarkers - depression/anxiety | Manual citation/reference screening | Observational primary study | 4 | 2 | 3 | 9 | Low | Large cohort/MR study; strong covariate adjustment but causal interpretation remains cautious. |
| S45 | Combined serum IL-6, C-reactive protein, and cortisol may distinguish patients with anhedonia in major depressive disorder | Systemic inflammatory biomarkers - depression | Manual citation/reference screening | Observational primary study | 4 | 1 | 3 | 8 | Low | Primary biomarker study; covariate adjustment scored according to reported methods. |
| S46 | Combining S100B and cytokines as neuro-inflammatory biomarkers for diagnosing generalized anxiety disorder: A proof-of-concept study based on machine learning | Systemic inflammatory biomarkers - anxiety | Manual citation/reference screening | Observational primary study | 4 | 1 | 3 | 8 | Low | GAD-focused primary biomarker study; proof-of-concept design. |
| S47 | Salivary inflammatory biomarkers as a predictor of post-traumatic stress disorder and depressive symptom severity in trauma patients: A prospective study | Salivary inflammatory biomarkers | Manual citation/reference screening | Observational primary study | 4 | 2 | 3 | 9 | Low | Prospective salivary biomarker study; PTSD classified separately as trauma/stress-related. |
| S48 | Oral microbiome composition is associated with depressive symptoms during pregnancy | Oral microbiome | Manual citation/reference screening | Observational primary study | 4 | 1 | 3 | 8 | Low | Pregnancy-specific population; hormone/oral-health confounding noted. |
| S49 | Stress and mental health symptoms in early pregnancy are associated with the oral microbiome | Oral microbiome | Manual citation/reference screening | Observational primary study | 4 | 2 | 3 | 9 | Low | Pregnancy cohort; includes relevant behavioral/oral-health covariates. |
| S50 | Oral microbiota among treatment-naïve adolescents with depression: A case-control study | Oral microbiome | Manual citation/reference screening | Observational primary study | 4 | 1 | 3 | 8 | Low | Treatment-naïve adolescents reduce medication/xerostomia confounding. |
| S51 | Dissecting the association between inflammation, depression and anxiety: Findings from the NHANES cohort and Mendelian randomization analysis | Systemic inflammatory biomarkers - depression/anxiety | Manual citation/reference screening | Observational primary study | 4 | 2 | 3 | 9 | Low | Large cohort/MR study; interpreted without treating IL-6 as a standardized clinical cutoff. |
| S52 | Increased inflammation predicts nine-year change in major depressive disorder diagnostic status | Systemic inflammatory biomarkers - depression | Manual citation/reference screening | Observational primary study | 4 | 2 | 3 | 9 | Low | Longitudinal evidence; supports early-warning interpretation. |
| S53 | Inflammatory markers and incident depression: Evidence in a population-based prospective study | Systemic inflammatory biomarkers - depression | Manual citation/reference screening | Observational primary study | 4 | 2 | 3 | 9 | Low | Population-based prospective study; useful for temporal evidence. |

# Supplementary Table S3. Included primary studies.

The included-study dataset comprises 53 primary human studies: 43 identified through database searching and 10 identified through citation/reference screening.

| **Study ID** | **Title** | **DOI** | **Evidence track** | **Source** |
| --- | --- | --- | --- | --- |
| S1 | Association between oral microbiome and depression: A population-based study | https://doi.org/10.1016/j.jad.2025.03.018 | Oral microbiome | Database search |
| S2 | Associations Between Oral Microbiota Pathogens and Elevated Depressive and Anxiety Symptoms in Men | 10.1155/da/9961595 | Oral microbiome | Database search |
| S3 | Oral microbiome composition, but not diversity, is associated with adolescent anxiety and depression symptoms | 10.1016/j.physbeh.2020.113126 | Oral microbiome | Database search |
| S4 | Probing the oral-brain connection: oral microbiome patterns in a large community cohort with anxiety, depression, and trauma symptoms, and periodontal outcomes | 10.1038/s41398-024-03122-4 | Oral microbiome | Database search |
| S5 | Relationship between depression and oral microbiome diversity: analysis of NHANES data (2009-2012) | 10.1186/s12903-025-06274-x | Oral microbiome | Database search |
| S6 | The association of depression and oral microbiota | 10.1038/s41598-025-26526-8 | Oral microbiome | Database search |
| S7 | Variations in the oral microbiome are associated with depression in young adults | 10.1038/s41598-021-94498-6 | Oral microbiome | Database search |
| S8 | Altered levels of salivary cytokines in patients with major depressive disorder | 10.1016/j.clineuro.2022.107390 | Salivary inflammatory biomarkers | Database search |
| S9 | Altered Serum IL-6 and TGF-β1 Levels Are Associated With Generalized Anxiety Disorder: A Case-Control Study | 10.1002/dneu.23004 | Systemic inflammatory biomarkers - anxiety | Database search |
| S10 | Altered serum interleukin-17A and interleukin-23A levels may be associated with the pathophysiology and development of generalized anxiety disorder | 10.1038/s41598-024-66131-9 | Systemic inflammatory biomarkers - anxiety | Database search |
| S11 | Anxiety disorders and CRP in a population cohort study with 54,326 participants: The LifeLines study | 10.1080/15622975.2018.1433325 | Systemic inflammatory biomarkers - anxiety | Database search |
| S12 | Association of interleukin-2 and interleukin-10 with the pathophysiology and development of generalized anxiety disorder: a case-control study | 10.1186/s12888-024-05911-z | Systemic inflammatory biomarkers - anxiety | Database search |
| S13 | Generalized Anxiety Disorder is Prospectively Associated With Decreased Levels of Interleukin-6 and Adiponectin Among Individuals from the Community | 10.1016/j.jad.2020.03.123 | Systemic inflammatory biomarkers - anxiety | Database search |
| S14 | A novel biomarker of interleukin 6 activity and clinical and cognitive outcomes in depression | 10.1016/j.psyneuen.2024.107008 | Systemic inflammatory biomarkers - depression | Database search |
| S15 | Altered serum TNF-α and MCP-4 levels are associated with the pathophysiology of major depressive disorder: A case-control study results | 10.1371/journal.pone.0294288 | Systemic inflammatory biomarkers - depression | Database search |
| S16 | Altered serum interleukin-7 and interleukin-10 are associated with drug-free major depressive disorder | 10.1177/2045125320916655 | Systemic inflammatory biomarkers - depression | Database search |
| S17 | Altered serum levels of TNF-α, IL-6, and IL-18 in depressive disorder patients | 10.1002/hup.2588 | Systemic inflammatory biomarkers - depression | Database search |
| S18 | Association between C-reactive protein (CRP) with depression symptom severity and specific depressive symptoms in major depression | 10.1016/j.bbi.2017.02.020 | Systemic inflammatory biomarkers - depression | Database search |
| S19 | Association between inflammatory cytokines and symptoms of major depressive disorder in adults | 10.3389/fimmu.2023.1110775 | Systemic inflammatory biomarkers - depression | Database search |
| S20 | Associations of number and severity of depressive episodes with C-reactive protein and Interleukin-6 | 10.1016/j.ajp.2017.02.016 | Systemic inflammatory biomarkers - depression | Database search |
| S21 | Cytokine-Related Genes and Inflammatory Profiles as Potential Biomarkers in Major Depressive Disorder | 10.30773/pi.2025.0013 | Systemic inflammatory biomarkers - depression | Database search |
| S22 | Evaluation of Serum Interleukin-6 and C-reactive Protein Levels in Drug-naive Major Depressive Disorder Patients | 10.7759/cureus.3868 | Systemic inflammatory biomarkers - depression | Database search |
| S23 | Evaluation of serum interleukin-1 receptor antagonist levels in major depressive disorder: A case-control study | 10.1002/hsr2.1175 | Systemic inflammatory biomarkers - depression | Database search |
| S24 | Evaluation of serum interleukin-12 and interleukin-4 as potential biomarkers for the diagnosis of major depressive disorder | 10.1038/s41598-024-51932-9 | Systemic inflammatory biomarkers - depression | Database search |
| S25 | Faster speed of onset of the depressive episode is associated with lower cytokine serum levels (IL-2,-4,-6,-10, TNF-α and IFN-γ) in patients with major depression | 10.1016/j.jpsychires.2021.06.033 | Systemic inflammatory biomarkers - depression | Database search |
| S26 | Higher levels of serum IL-1β and TNF-α are associated with an increased probability of major depressive disorder | 10.1016/j.psychres.2020.113568 | Systemic inflammatory biomarkers - depression | Database search |
| S27 | Higher serum interleukin-12 levels are associated with the pathophysiology of major depressive disorder: A case-control study results | 10.1002/hsr2.1005 | Systemic inflammatory biomarkers - depression | Database search |
| S28 | Inflammatory cytokines, complement factor H and anhedonia in drug-naïve major depressive disorder | 10.1016/j.bbi.2021.03.022 | Systemic inflammatory biomarkers - depression | Database search |
| S29 | Pro- and anti-inflammatory cytokines, but not CRP, are inversely correlated with severity and symptoms of major depression | 10.1016/j.psychres.2016.02.052 | Systemic inflammatory biomarkers - depression | Database search |
| S30 | Prospective associations between hsCRP and GlycA inflammatory biomarkers and depression: The Brazilian longitudinal study of adult health (ELSA-Brasil) | https://doi.org/10.1016/j.jad.2020.03.074 | Systemic inflammatory biomarkers - depression | Database search |
| S31 | Serum CC Chemokines as Potential Biomarkers for the Diagnosis of Major Depressive Disorder | 10.2147/PRBM.S384267 | Systemic inflammatory biomarkers - depression | Database search |
| S32 | Serum FAM19A5 levels: A novel biomarker for neuroinflammation and neurodegeneration in major depressive disorder | 10.1016/j.bbi.2020.03.021 | Systemic inflammatory biomarkers - depression | Database search |
| S33 | Serum levels of interleukin-33 and mesencephalic astrocyte derived neurotrophic factors in patients with major depressive disorder: a cross-sectional comparative design | 10.1186/s12888-023-05463-8 | Systemic inflammatory biomarkers - depression | Database search |
| S34 | Suppressors of Cytokine Signaling Are Decreased in Major Depressive Disorder Patients | 10.3390/jpm12071040 | Systemic inflammatory biomarkers - depression | Database search |
| S35 | The relationship between serum resolvin D1, NLRP3, cytokine levels, and adolescents with first-episode medication-naïve major depressive disorder | 10.1186/s12888-024-05724-0 | Systemic inflammatory biomarkers - depression | Database search |
| S36 | The relationship of peripheral blood cell inflammatory biomarkers and psychological stress in unmedicated major depressive disorder | 10.1016/j.jpsychires.2024.06.013 | Systemic inflammatory biomarkers - depression | Database search |
| S37 | Youth depression and inflammation: Cross-sectional network analyses of C-Reactive protein, interleukin-6 and symptoms in a population-based sample | 10.1016/j.jpsychires.2022.03.065 | Systemic inflammatory biomarkers - depression | Database search |
| S38 | Cytokine profile in first-episode drug-naive major depressive disorder patients with or without anxiety | 10.1186/s12888-024-05536-2 | Systemic inflammatory biomarkers - depression/anxiety | Database search |
| S39 | Inflammation, depression, and anxiety disorder: A population-based study examining the association between Interleukin-6 and the experiencing of depressive and anxiety symptoms | 10.1016/j.psychres.2020.112809 | Systemic inflammatory biomarkers - depression/anxiety | Database search |
| S40 | New Insights into the oral microbiota and host epigenetic changes in obsessive compulsive disorder and major depressive disorder: Focus on BDNF | 10.1016/j.phrs.2025.107891 | Oral microbiome | Database search |
| S41 | HIGH-SENSITIVITY C-REACTIVE PROTEIN, POSSIBLE BIOMARKER FOR DEPRESSION IN ELDERLY POPULATION | 10.4183/aeb.2019.215 | Systemic inflammatory biomarkers - depression | Database search |
| S42 | High levels of TNF-α are associated with symptoms of depression in health professionals at a hospital | 10.1016/j.rpsm.2021.10.002 | Systemic inflammatory biomarkers - depression | Database search |
| S43 | Serum cytokines-based biomarkers in the diagnosis and monitoring of therapeutic response in patients with major depressive disorder | 10.1016/j.intimp.2023.110108 | Systemic inflammatory biomarkers - depression | Database search |
| S44 | Role of inflammation in depression and anxiety: Tests for disorder specificity, linearity and potential causality of association in the UK Biobank | 10.1016/j.eclinm.2021.100992 | Systemic inflammatory biomarkers - depression/anxiety | Manual citation/reference screening |
| S45 | Combined serum IL-6, C-reactive protein, and cortisol may distinguish patients with anhedonia in major depressive disorder | 10.3389/fnmol.2022.935031 | Systemic inflammatory biomarkers - depression | Manual citation/reference screening |
| S46 | Combining S100B and cytokines as neuro-inflammatory biomarkers for diagnosing generalized anxiety disorder: A proof-of-concept study based on machine learning | 10.3389/fpsyt.2022.881241 | Systemic inflammatory biomarkers - anxiety | Manual citation/reference screening |
| S47 | Salivary inflammatory biomarkers as a predictor of post-traumatic stress disorder and depressive symptom severity in trauma patients: A prospective study | 10.1016/j.bbi.2024.05.011 | Salivary inflammatory biomarkers | Manual citation/reference screening |
| S48 | Oral microbiome composition is associated with depressive symptoms during pregnancy | 10.1016/j.bbih.2025.100978 | Oral microbiome | Manual citation/reference screening |
| S49 | Stress and mental health symptoms in early pregnancy are associated with the oral microbiome | 10.1136/bmjment-2024-301100 | Oral microbiome | Manual citation/reference screening |
| S50 | Oral microbiota among treatment-naïve adolescents with depression: A case-control study | 10.1016/j.jad.2025.01.000 | Oral microbiome | Manual citation/reference screening |
| S51 | Dissecting the association between inflammation, depression and anxiety: Findings from the NHANES cohort and Mendelian randomization analysis | 10.1038/s41380-020-00968-4 | Systemic inflammatory biomarkers - depression/anxiety | Manual citation/reference screening |
| S52 | Increased inflammation predicts nine-year change in major depressive disorder diagnostic status | 10.1037/abn0000716 | Systemic inflammatory biomarkers - depression | Manual citation/reference screening |
| S53 | Inflammatory markers and incident depression: Evidence in a population-based prospective study | 10.1016/j.psyneuen.2022.105806 | Systemic inflammatory biomarkers - depression | Manual citation/reference screening |

# Supplementary Table S4. PRISMA support counts and full-text exclusion reasons.

Part A. PRISMA count support.

| **Stage** | **Count** | **Notes** |
| --- | --- | --- |
| Records identified from databases | 1848 | Sum of PubMed, Web of Science, and PsycINFO search blocks |
| Reports identified from citation/reference screening | 10 | Additional eligible records identified by manual citation/reference screening |
| Duplicate records removed before screening | 625 | Duplicates removed from database-derived records before title/abstract screening |
| Records screened | 1223 | Unique database-derived records screened by title and abstract |
| Records excluded at title/abstract screening | 1151 | Excluded for non-human studies, review/meta-analysis records, no relevant psychiatric outcome, gut-only microbiome focus, or no relevant inflammatory/oral microbiome outcome |
| Reports sought for retrieval from database searching | 72 | Potentially eligible database-derived reports advanced to full-text review |
| Reports not retrieved from database searching | 0 | All database-derived reports were retrieved for full-text review |
| Reports assessed for eligibility from database searching | 72 | Database-derived full-text reports assessed |
| Reports excluded after full-text review from database searching | 29 | See Full-text Exclusion Reasons sheet |
| Reports sought for retrieval from citation/reference screening | 10 | Potentially eligible reports identified through reference-list screening |
| Reports not retrieved from citation/reference screening | 0 | All citation-derived reports were retrieved |
| Reports assessed for eligibility from citation/reference screening | 10 | Citation-derived full-text reports assessed |
| Reports excluded after full-text review from citation/reference screening | 0 | All citation-derived reports met eligibility criteria |
| Studies included from database searching | 43 | Included in the qualitative synthesis |
| Studies included from citation/reference screening | 10 | Included in the qualitative synthesis |
| Primary studies included in qualitative synthesis | 53 | 42 inflammatory/salivary biomarker studies and 11 oral microbiome studies |

Part B. Full-text exclusion reasons.

| **Reason for exclusion** | **n** | **Notes** |
| --- | --- | --- |
| Review/meta-analysis or context-only secondary source | 11 | Not counted as primary included studies; used only for background or contextual discussion when appropriate |
| Tangential intervention study or insufficient relevance to the review question | 8 | Did not directly address eligible psychiatric-biomarker or psychiatric-oral microbiome associations in an extractable way |
| Outside the final review scope after full-text assessment | 6 | Inadequate alignment with the depression/anxiety plus biomarker/oral microbiome focus |
| Did not report an eligible psychiatric or biomarker/oral microbiome outcome in usable form | 4 | Insufficiently extractable for the qualitative synthesis |
| Total full-text exclusions from database searching | 29 | Matches the PRISMA count |

Part C. Source summary for included studies.

| **Source category** | **n** |
| --- | --- |
| Database search included studies | 43 |
| Citation/reference-screened included studies | 10 |
| Total included studies | 53 |
| Inflammatory/salivary biomarker studies | 42 |
| Oral microbiome studies | 11 |
